# Task-adaptive physical reservoir computing

**DOI:** 10.1038/s41563-023-01698-8

**Published:** 2023-11-13

**Authors:** Oscar Lee, Tianyi Wei, Kilian D. Stenning, Jack C. Gartside, Dan Prestwood, Shinichiro Seki, Aisha Aqeel, Kosuke Karube, Naoya Kanazawa, Yasujiro Taguchi, Christian Back, Yoshinori Tokura, Will R. Branford, Hidekazu Kurebayashi

**Affiliations:** 1 London Centre for Nanotechnology, University College London, London, UK; 2 Blackett Laboratory, Imperial College London, London, UK; 3 Department of Applied Physics, University of Tokyo, Tokyo, Japan; 4 Physik-Department, Technische Universität München, Garching, Germany; 5 Munich Center for Quantum Science and Technology (MCQST), Munich, Germany; 6 RIKEN Center for Emergent Matter Science (CEMS), Wako, Japan; 7 Tokyo College, University of Tokyo, Tokyo, Japan; 8 London Centre for Nanotechnology, Imperial College London, London, UK; 9 Department of Electronic and Electrical Engineering, University College London, London, UK; 10 WPI Advanced Institute for Materials Research, Tohoku University, Sendai, Japan

**Keywords:** Electronic devices, Magnetic properties and materials, Magnetic properties and materials

## Abstract

Reservoir computing is a neuromorphic architecture that may offer viable solutions to the growing energy costs of machine learning. In software-based machine learning, computing performance can be readily reconfigured to suit different computational tasks by tuning hyperparameters. This critical functionality is missing in ‘physical’ reservoir computing schemes that exploit nonlinear and history-dependent responses of physical systems for data processing. Here we overcome this issue with a ‘task-adaptive’ approach to physical reservoir computing. By leveraging a thermodynamical phase space to reconfigure key reservoir properties, we optimize computational performance across a diverse task set. We use the spin-wave spectra of the chiral magnet Cu_2_OSeO_3_ that hosts skyrmion, conical and helical magnetic phases, providing on-demand access to different computational reservoir responses. The task-adaptive approach is applicable to a wide variety of physical systems, which we show in other chiral magnets via above (and near) room-temperature demonstrations in Co_8.5_Zn_8.5_Mn_3_ (and FeGe).

## Abstract (web summary)

Current physical neuromorphic computing faces critical challenges of how to reconfigure key physical dynamics of a system to adapt computational performance to match a diverse range of tasks. Here the authors present a task-adaptive approach to physical neuromorphic computing based on on-demand control of computing performance using various magnetic phases of chiral magnets.

## Main

Physical separation between processing and memory units in conventional computer architectures causes substantial energy waste due to the repeated shuttling of data, known as the von Neumann bottleneck. To circumvent this, neuromorphic computing^1,2^, which draws inspiration from the brain to provide integrated memory and processing, has attracted a great deal of attention as a promising future technology. Reservoir computing^3–5^ is a type of neuromorphic architecture with complex recurrent pathways (the ‘reservoir’) that maps input data to a high-dimensional space. Weights within the reservoir are randomly initialized and fixed, and only the small one-dimensional weight vector that connects the reservoir to the output requires optimization using computationally cheap linear regression. As such, reservoir computing can achieve powerful neuromorphic computation at a fraction of the processing cost relative to other schemes, for example, deep neural networks, where the whole weight network (typically involving more than millions of nodes) must be trained^6^.

Although reservoir computing was originally conceived in software^3^, nonlinear and history-dependent responses of physical systems have also been exploited as reservoirs^7,8^. The field of physical reservoir computing has been rapidly expanding with several promising demonstrations using optical systems^9^, analogue electronic circuits^10^, memristors^11,12^, ferroelectrics^13^, magnetic systems^14–19^ and even a bucket of water^20^. Skyrmions, topologically non-trivial magnetic whirls, have also been proposed as hosts for reservoir computing^21–24^ and experimentally demonstrated^25–27^ as part of rapidly growing research efforts towards neuromorphic computing using magnetic systems^28–32^.

Despite such rapid development, one of the outstanding challenges for creating powerful physical reservoirs is establishing a methodology for task-adaptive control of reservoir properties^8^, often characterized by the nonlinearity, memory capacity and complexity metrics of the reservoir^33–36^. However, physical systems typically have a narrow and fixed set of reservoir properties without having much room to change, since the above metrics tend to be constrained to a particular response of a physical system. Because of this, a physical reservoir tends to perform well for some specific tasks but poorly for others, which require different reservoir properties. This is a severe drawback relative to software reservoirs, where such properties can be tuned by changing lines of code.

Here, we demonstrate task-adaptive reservoir computing using the spectral space of a physical system that has rich, phase-tunable dynamical modes. As a model system of this approach, we use spin resonances of the chiral magnet Cu_2_OSeO_3_ (refs. ^37–39^). Since different magnetic phases (skyrmion, helical and conical phases) exhibit distinct resonances, they offer broadly varying reservoir properties and computing performance, which can be reconfigurably tuned via the magnetic field and temperature. We use magnetic field cycling^40,41^ to input data and measure the spin-wave spectra at each input step to efficiently achieve high-dimensional mapping. By quantitatively assessing each phase as a reservoir, we find that the thermodynamically metastable skyrmion phase has a strong memory capacity due to the magnetic field-driven gradual nucleation of skyrmions with an excellent performance in future prediction tasks. By contrast, the conical phase has modes with great reservoir nonlinearity and complexity, which are ideal for transformation tasks. By making full use of this phase-tunable nature, we achieve a strong performance across a broad range of tasks in a single physical system. High-temperature demonstration of the task-adaptive physical reservoir concept using other chiral magnets, that is, Co_8.5_Zn_8.5_Mn_3_ and FeGe, indicates that the concept is indeed ubiquitous.

## Reservoir computing scheme

**Fig. 1:**
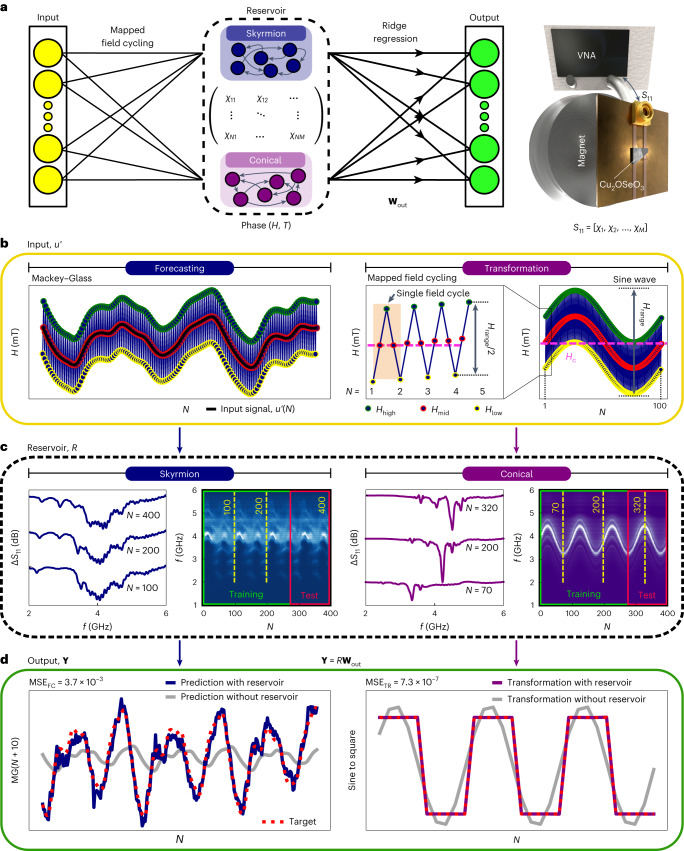
Working principles of physical reservoir computing with a chiral magnet. **a**, Illustration of a task-adaptive reservoir computing framework. Different magnetic phases are accessed by controlling the external magnetic field (*H*) and temperature (*T*). The rightmost panel shows the experimental schematic of the VNA-assisted spin-wave spectroscopy setup. VNA, vector network analyser. **b**, Typical input scheme for forecasting (left: Mackey–Glass signal) and transformation (right: sine wave) tasks. The original input signal, *u*(*t*), is mapped to *u'*(*N*), which is defined by the mapped field-cycling protocol (see main text and Methods for details). Note that *H*_range_ defines the full range of applied fields, where the distance between *H*_low_ and *H*_high_ at any given *N*, is the width of cycling, *H*_range_/2. A single field cycle is highlighted by the orange box in the transformation panel. **c**, *S*_11_ (denoted as Δ*S*_11_ after pre-processing; see Methods) as a function of the frequency *f* after accumulating *N* field cycles and visualization of *R*(*N*, *M*); a collective spectral evolution for *N* field cycles for skyrmion and conical phases, separated into ‘training’ and ‘test’ datasets. **d**, Results after applying **W**_out_ on the unseen ‘test’ dataset. Left: forecasting of a differential chaotic time-series data, Mackey–Glass signal by 10 future steps. Right: transformation of a sine wave to a square-wave signal. In both cases, reservoir prediction (transformation) results are plotted in blue (purple), the red dotted line depicts the target signal and the grey line represents the control prediction where ridge regression is performed on the raw input data without the physical reservoir. MSE_FC_ and MSE_TR_ quantify the computation performance of forecasting and transformation, respectively.

Our physical reservoir (Fig. 1a) is constructed using the field- and temperature-dependent gigahertz spin dynamics of Cu_2_OSeO_3_ (ref. ^38^). We apply a specific sequence of magnetic field inputs and map out the spin-wave spectra of Cu_2_OSeO_3_ to form a two-dimensional matrix^18^. Subsequently, the reservoir matrix is multiplied by a weight vector **W**_out_ to produce the individual output value for each input. We use standard ridge regression to optimize **W**_out_ for each task with training data. The trained reservoir is then run for the unseen test datasets to assess the reservoir computing performance via the mean squared error (MSE; see Methods). The rich phase diagram of Cu_2_OSeO_3_ offers multiple magnetic textural phases, including the thermodynamically metastable skyrmion phase^40–43^, each of which exhibits distinct spin dynamics properties. The task-adaptive nature of our physical reservoir comes from the reconfigurable on-demand control between these magnetic phases via both temperature and magnetic field.

As shown in Fig. 1b, the input layer consists of sequential magnetic field values, *u*′ = (*H*_1_, *H*_2_, *H*_3_, …, *H*_*n*_), produced by projecting a given input function of each task onto the field. Taking the transformation task as an example, each field cycle *N* starts with a low magnetic field *H*_low_, increasing to a high magnetic field *H*_high_ and comes back to a new *H*_low_, where their separation is defined by *H*_range_/2 with a centre field *H*_c_. The individual field points (*H*_low_, *H*_mid_ and *H*_high_) are modulated by the input functions tailored for specific tasks. For the transformation tasks, the input function is a sine curve encoded over 100 field cycles; our forecasting tasks use a chaotic oscillatory Mackey–Glass time series^44^ to modulate the field-cycling base with *N*, as shown in the left panel of Fig. 1b (Methods). This scheme can be applied to input any time-series dataset into the physical reservoir.

To create a two-dimensional reservoir matrix, *R*(*N*, *M*), we use an experimental setup illustrated in the rightmost panel of Fig. 1a to measure the microwave reflection spectra *S*_11_ of Cu_2_OSeO_3_ crystals, recording *M* frequency channels between 1 and 6 GHz for each field cycle at *H*_low_ labelled by *N* (Methods). Using this scheme, the physical reservoir effectively broadcasts a single field input value to multiple *M* values as frequency multiplexing. Figure 1c shows the spectral output of our reservoir in response to input time-series datasets (left: Mackey–Glass, right: sine wave). The spectral states of each phase (left: skyrmion, right: conical) change as we perform field cycling—see the individual spectra sampled at different *N* values in Fig. 1c. Using *S*_11_(*N*, *f*) in the colour heatmap plots, we form *R*(*N*, *M*) as shown in the middle panel of Fig. 1a, where *χ*_*ij*_ represents the magnetic susceptibility for each input field and frequency.

Using 70% of the reservoir response as the training dataset *R*_train_ shown in Fig. 1c, we perform ridge regression to calculate the weights **W**_out_ against a target function **Y**, where **Y** = *R*_train_**W**_out_. The calculated **W**_out_ and the remaining 30% of the reservoir *R*_test_ are subsequently used to evaluate the reservoir performance via the MSE. Figure 1d exemplifies this final process of our reservoir computing protocol by showing the physical reservoir’s attempt (blue line) at reproducing the target signal (red dotted line) for two tasks: the left panel shows a forecast of the chaotic Mackey–Glass signal ten future steps ahead (MG(*N* + 10)); the right panel shows the nonlinear transformation of a sine-wave input to a square-wave target. For both tasks, the excellent performance of reservoir computing is confirmed by the low MSE values: 3.7 × 10^−3^ for the forecasting task by the skyrmion reservoir; 7.3 × 10^−7^ for the transformation task by the conical reservoir. The virtue of the reservoir components can be assessed using these two values compared with those calculated by computing the same tasks without the reservoirs (grey curves): 6.2 × 10^2^ and 5.4 × 10^2^ for the forecasting and transformation tasks, respectively.

## Phase-tunable physical reservoir computing

**Fig. 2:**
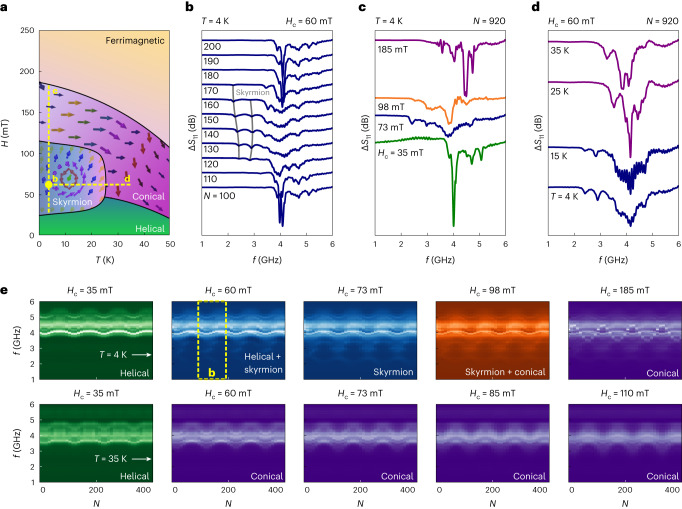
Field-cycling-dependent spin-wave spectra as a physical reservoir. **a**, Schematic of the temperature phase diagram for the bulk crystal Cu_2_OSeO_3_. The yellow dashed vertical (horizontal) line indicates the experimental conditions for our cycling experiments shown in **c** (**d**). **b**, The cycling number dependence of the spin-wave spectra in Cu_2_OSeO_3_ for *H*_c_ = 60 mT and 4 K. The evolution of the skyrmion-phase spectrum is shown for increasing values of *N*. Grey lines are added as a guide to the eye to keep track of the skyrmion modes. **c**, *H*_c_ dependence of the spin-wave spectra in Cu_2_OSeO_3_ for 4 K after 920 field cycles. **d**, Temperature dependence of the spin-wave spectra for *H*_c_ = 60 mT after 920 field cycles. **e**, Microwave absorption spectra as a function of *f* and *N* for different values of *H*_c_ at *T* = 4 K (upper row) and 35 K (lower row). The input signal in all plots is a sine wave with *H*_range_ = 90 mT.

The phase-tunable nature of our physical reservoir computing stems from the rich magnetic phase diagram of Cu_2_OSeO_3_ shown in Fig. 2a ref. ^37^. Added to this diagram is the metastable skyrmion phase, which can be generated at low temperatures below ~35 K using quenching techniques or field-cycling protocols^40–42^. We leverage this phase tunability to create the task-adaptive nature of our physical reservoir, as detailed below.

Figure 2b displays the *N* dependence of the spectra for *H*_c_ and temperature inside the skyrmion phase. For *N* = 100, a sharp peak around 4 GHz can be clearly observed, corresponding to low-energy spin-wave modes of the thermodynamically stable conical phase^39–41^. As we cycle further, the conical mode amplitude shrinks and the skyrmion modes appear around 2–3 GHz, as highlighted by the grey curves for *N* = 130–170. These are the counterclockwise and breathing modes of the metastable low-temperature skyrmion phase generated by field cycling^40,41^. The mode frequencies move with our input magnetic fields, and as the cycling proceeds the skyrmions are continuously destroyed and renucleated, as evident from the peak amplitude. When we carry out experiments for different *H*_c_ values, we can clearly demonstrate the tunability of the magnetic phases for our reservoir computing, as shown in Fig. 2c where the spectra are obtained after 920 field cycles with *H*_range_ = 90 mT at 4 K. A similar tunability can be achieved by changing the temperature at a fixed *H*_c_ of 60 mT, as shown in Fig. 2d. The skyrmion modes are clearly identified for 4 K and 15 K and disappear for higher temperatures (25 K and 35 K), where the spectra are dominated by those from the conical phase. Finally, a collection showing the field-cycle evolution of spectra for various *H*_c_ and temperatures is presented in Fig. 2e to demonstrate the range of phase/spectral tunability. Individual spectral scans for further evolution of *N* as a variation of *H*_c_ can be found in Supplementary Fig. 3 (Supplementary Note 1).

## Reservoir performance

**Fig. 3:**
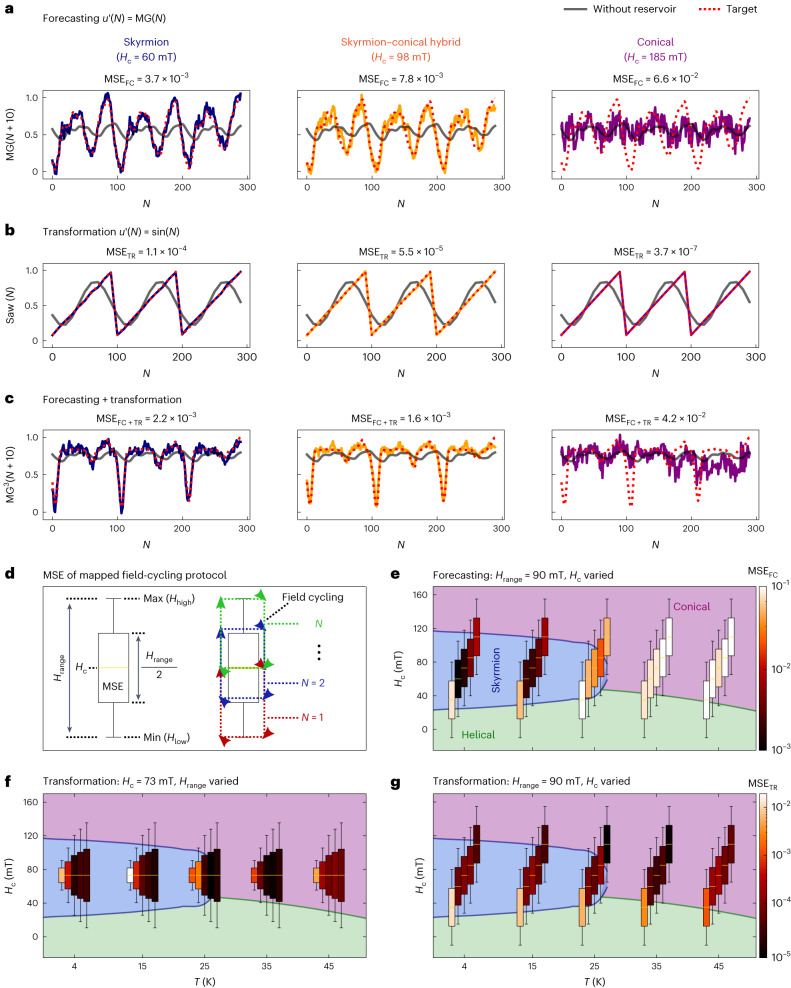
Reservoir computing performance of different magnetic phase spaces. MSE performance comparison of different computation tasks across three distinct physical phases (skyrmion, skyrmion–conical hybrid and conical) at *T* = 4 K. In **a**–**c**, the red dotted and grey curves represent the target functions and the computation results without the physical reservoirs, respectively. Blue, orange and purple curves display calculations with the physical reservoirs of skyrmion, skyrmion–conical hybrid and conical phases, respectively. **a**, Forecasting a Mackey–Glass chaotic time series of ten steps ahead (MG(*N* + 10)). **b**, Nonlinear transformation of a sine-wave input into saw waveforms. **c**, Combined transform/forecasting of ten future steps of a cubed Mackey–Glass signal. **d**, Illustration of the mapped field-cycling protocol visualized as a modified boxplot (details in the main text). **e**,**g**, Evaluation of MSE values at a constant *H*_range_ as a variation of *H*_c_ and *T*, respectively, for forecasting (MG(*N* + 10)) (**e**) and transformation (square wave) (**g**) target applications. **f**, Evaluation of MSE values at a constant *H*_c_ as a variation of *H*_range_ and *T* for a transformation (square wave) target application. The colour scale in **g** also applies to **f**.

Figure 3a–c compares the reservoir’s performance on different tasks using magnetic phases of skyrmion (*H*_c_ = 60 mT), skyrmion–conical hybrid (*H*_c_ = 98 mT) and conical modes (*H*_c_ = 185 mT) at 4 K with *H*_range_ = 90 mT and *N* = 1,000. For forecasting, the system is trained to predict the future behaviour of a Mackey–Glass signal of ten steps ahead. The reservoir performance is evaluated quantitatively by calculating the MSE between the reservoir prediction and the target signal.

As shown in Fig. 3a, when *H*_c_ is increased and the reservoir is transfigured from the skyrmion to the conical phase, the prediction performance deteriorates and the MSE increases by a factor of approximately 18. In the conical phase, the reservoir prediction is as bad as the one without the reservoir. The opposite trend is observed for transformation tasks, where the MSE is notably improved when switching from the skyrmion reservoir to the conical reservoir, as shown in Fig. 3b. Although the skyrmion reservoir still performs well with an MSE of the order of 10^−4^, the conical reservoir excels with an MSE of 3.7 × 10^−7^ for the sine-to-saw transformation task. By setting *H*_c_ at 98 mT, we create a hybrid reservoir phase where both skyrmion and conical modes coexist. This particular reservoir configuration outperforms both the individual skyrmion and conical reservoirs for a complex forecasting–transformation task of predicting ten future steps ahead for a cubed Mackey–Glass signal from a normal Mackey–Glass input shown in Fig. 3c. See Methods and Supplementary Note 3 for details of target generations and a broader selection of further forecasting and transformation tasks, with a tunable reservoir performance demonstrated throughout in Supplementary Fig. 6.

Figure 3e–g maps the observed reservoir performance on the phase diagram, with Fig. 3d as an aid to reading these plots. The upper and lower whiskers represent the maximum and minimum magnetic field values in the cycling scheme, respectively. The height of the box represents *H*_range_, and the central line defines *H*_c_. The MSE values are encoded as the box colour. The initial cycle begins at the bottom of the lower whisker and gradually moves up and down as a function of *N*. Figure 3e shows the reservoir performance for forecasting MG(*N* + 10) at *H*_range_ = 90 mT. The strongest performance is found when the field cycling lies entirely inside the skyrmion phase at lower temperatures. The performance gradually worsens as the field cycling moves beyond the skyrmion phase and is reduced dramatically when leaving the skyrmion phase at high temperatures. This excellent forecasting performance of the skyrmion reservoir is highly correlated with its memory capacity, as we discuss below.

For the transformation tasks, we show the reservoir performance for two parameter dependencies, *H*_range_ and *H*_c_. In Fig. 3f, where a variation of *H*_range_ for *H*_c_ = 73 mT is shown, it is clear for all measured temperatures that larger *H*_range_ values provide an optimal reservoir performance, maximizing the balance between the key reservoir properties associated with the tasks. In Fig. 3g, we observe that the reservoirs run with input mappings extending deeper into the helical phase (*H*_c_ = 35 mT) perform far more poorly, whereas the optimal performance for the transformation task is demonstrated when the reservoir substantially includes the conical phase that has strong nonlinearity and complexity. The MSE values displayed for Fig. 3e,g highlight that the performance from the identical reservoirs is starkly different between the two types of computational task.

**Fig. 4:**
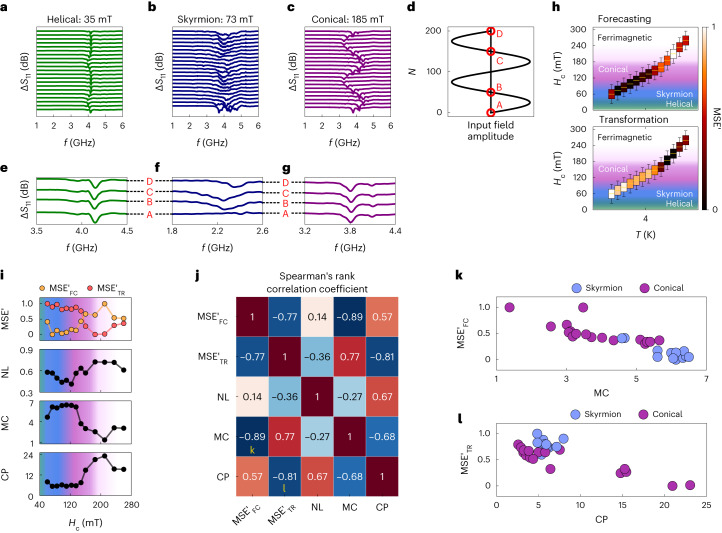
Computation properties associated with physical characteristics. **a**–**c**, Spin-wave spectra of the helical (**a**), skyrmion (**b**) and conical (**c**) magnetic phases. **d**, Sine-wave input sequence defining the applied field amplitudes. **e**–**g**, Spin-wave spectra of the helical (**e**), skyrmion (**f**) and conical (**g**) phases at nodes of the sine-wave input fields from **d**. **h**, *H*_c_ evolution of the MSE values at *T* = 4 K and *H*_range_ = 90 mT, for forecasting (MG(*N* + 10)) and transformation (square wave) target applications. Note that MSE' denotes the normalized scale of MSE for [0, 1], where 0 (1) represents the best (worst) MSE. A (meta)stable magnetic field range for each phase is colour-coded. **i**, MSE' and task-agnostic metric results as a function of *H*_c_ at *T* = 4 K. **j**, Correlation matrix of Spearman’s rank correlation coefficient. **k**, Performance of forecasting as an evolution of the MC metric. **l**, Performance of transformation as an evolution of the CP metric.

The computational performance of our magnetic reservoirs can be related to their physical properties. Figure 4a–c displays the spectral evolution of different magnetic phases with field cycling. The high (low) transformation performance of the conical (helical) phase can be associated with the size of the frequency shift by the magnetic field. The dispersion curve of the helical phase displays a notably flat profile in comparison with the other magnetic phases in chiral magnets^45^, resulting in a poor computational performance with its peak position shifting very weakly in response to the field input. Much higher amplitude frequency shifts are found in the highly performing conical and skyrmion phases, producing strong nonlinearity and complexity in their reservoirs, and hence low MSE values in the transformation tasks—see further/detailed analysis in Supplementary Note 2 and Supplementary Fig. 4. The origin of the excellent performance of the skyrmion reservoirs for forecasting tasks can be explained by comparing the spectra across the three phases at the same field values but at different points in the input field cycle, labelled as A–D in Fig. 4d. The spectra of both the helical (Fig. 4e) and conical (Fig. 4g) phases are identical across points A–D, showing that these phases respond only to the current field input being applied and lack any memory response for magnetic field inputs. By contrast, the skyrmion spectra in Fig. 4f are dissimilar across points A–D, meaning that the spectral response depends not on only the field value but also past field inputs. This is the source of the crucial physical memory response for forecasting tasks, arising from the magnetic field-driven nucleation of metastable skyrmions and the annihilation of other magnetic phases^40–43^. More quantitative and detailed discussions are available in the next section and Supplementary Note 2.

## Reservoir metrics

To gain further insights into our reservoir properties, we use task-agnostic reservoir metrics, that is nonlinearity (NL), memory capacity (MC) and complexity (CP)^34,35^, to characterize the reservoir properties (see Supplementary Note 4 for details) as well as correlation between these metrics and the normalized MSE for the forecasting (transformation) task (${{{{\rm{MSE}}}}}_{{{{\rm{FC}}}}({{{\rm{TR}}}})}^{{\prime} }$). We performed both forecasting and transformation tasks across a wide range of *H*_c_ values at 4 K, as shown in Fig. 4h. In parallel, the metric scores were evaluated for each *H*_c_, as plotted in Fig. 4i. It is clear that the MC metric shows similar behaviour to the performance of ${{{{\rm{MSE}}}}}_{{{{\rm{FC}}}}}^{{\prime} }$ with *H*_c_, i.e., forecasting performance and MC increases then decreases with *H*_c_, suggesting that MC is a key property for a better performance in forecasting tasks. As discussed earlier, MC in the skyrmion phase stems from the history-dependent fading memory property generated by its gradual skyrmion nucleation with repeated field cycles^40,41^. Rich and complex spin-wave mode dispersion in the conical/ferrimagnetic phases provides the physical basis for the high NL and CP scores, offering a strong transformation task performance (see more detailed discussions in Supplementary Note 2).

The correlation between different parameters can be numerically discussed using the standard Spearman’s rank correlation coefficient^46^ as shown in Fig. 4j (Methods). Here, the algorithm outputs [−1, 1], where 1 (−1) corresponds to perfect proportionality (inverse proportionality) and 0 denotes no correlation. Note that since the better performance in each task is represented by a lower MSE′, the correlation with a negative value to each metric indicates a positive correlation in our analysis. The performance of time series forecasting strongly correlates with MC (−0.89) and CP (0.57), revealing that MC (CP) is favoured (disfavoured) for these particular types of task, while the opposite is true for transformation tasks. It is also important to highlight that MC and CP have a clear negative correlation (−0.68), indicating the trade-off nature between these two reservoir properties. Subsequently, the high correlation between NL and CP (0.67) suggests that a more nonlinear system enhances the amount of meaningful input data encoded in the reservoir to display high complexity.

We show the specific relationship between the reservoir performance evaluated by MSE′ and MC (CP) as plotted in Fig. 4k (4l), where the colour of the dots indicates which magnetic phase the metrics were evaluated against. Following the Spearman’s rank correlation values for each pair, both plots show a negative trend for each reservoir characteristic. Unlike the conical phase, the metrics of the skyrmion phase appear to be clustered around high values of MC between 4 and 7, further confirming that the skyrmion reservoir’s memory is responsible for the excellent forecasting performance. By contrast, Fig. 4l shows that the system’s ability to perform transformation tasks reaches its full potential by maximizing the complexity, which occurs when the conical phase dominates the magnet—see further discussions on the reservoir metrics and other correlations in Supplementary Fig. 7 (Supplementary Note 5).

## Above-room-temperature demonstration

**Fig. 5:**
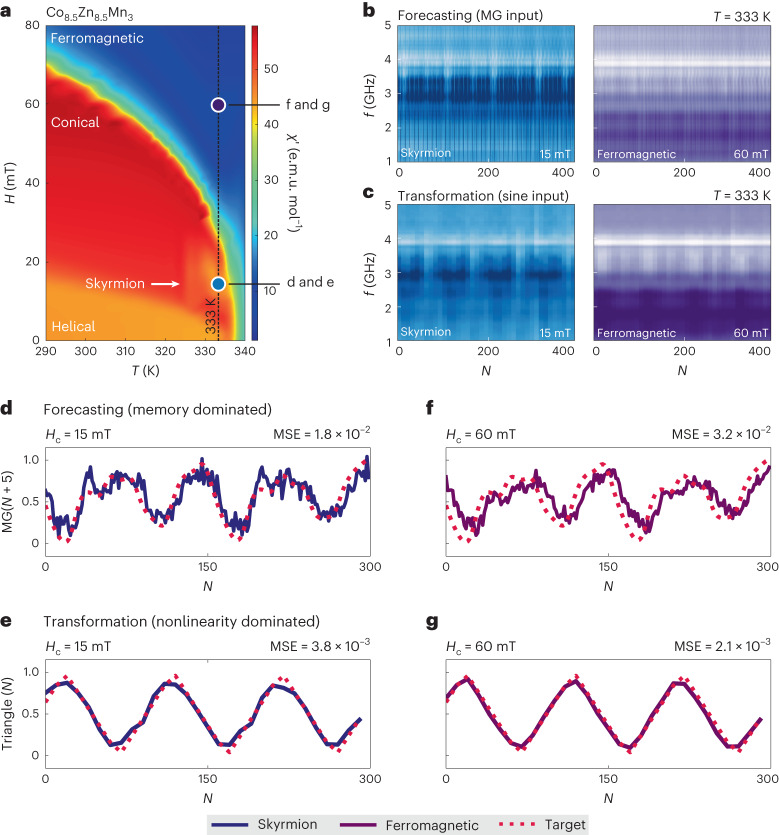
Above-room-temperature demonstration of task adaptability using Co_8.5_Zn_8.5_Mn_3_. **a**, Two-dimensional plot of the real part of the a.c. susceptibility (*χ*′) to identify magnetic phases in a Co_8.5_Zn_8.5_Mn_3_ crystal with helical, skyrmion, conical and ferromagnetic phases. The vertical dotted line represents the temperature at which we performed the reservoir computing experiments. **b**,**c**, Spin dynamics spectra measured during field cycling *N* showing the reservoir computing performance for forecasting (**b**) and transformation (**c**) at 333 K, at different centre fields of 15 mT (left spectra) and 60 mT (right spectra). **d**–**g**, Reservoir computing performance of predicting the nonlinear Mackey–Glass function for five future steps (**d**,**f**) and transformation from a sine input signal to triangle output function (**e**,**g**). The red dotted curves denote the target function and the blue and purple solid curves are calculations generated via our reservoir computing.

Finally, we present that the task-adaptive reservoir concept can be transferable to different material systems, here using other chiral magnets: Co_8.5_Zn_8.5_Mn_3_ (Fig. 5) and FeGe (see Supplementary Note 7 and Supplementary Fig. 9). Consistent with earlier work on the same class of Co-Zn-Mn alloy materials (for example, refs. ^47,48^), multiple magnetic phases in Co_8.5_Zn_8.5_Mn_3_ can be clearly recognized in the plot of alternating-current (a.c.) susceptibility measurements shown in Fig. 5a. In particular, in the vicinity of its Curie temperature, we can recognize the signature of a thermodynamically stable skyrmion phase—see also Supplementary Fig. 8 (Supplementary Note 6) for the imaginary part of the a.c. susceptibility to highlight this phase. We therefore performed reservoir computing schemes at 333 K with different magnetic centre fields *H*_c_ = 15 and 60 mT with a 10 mT cycling width. In Fig. 5b,c, we show the spectra of magnetic resonance during field cycling of both the nonlinear Mackey–Glass and sine input functions to carry out the future prediction and transformation tasks, respectively. For both tasks, we observe that the spectra depend strongly on the centre field, demonstrating the phase tunability of physical reservoirs in this material. Using these physical reservoirs with different magnetic phases, we carried out both tasks, the results of which are displayed in Fig. 5d–g. For the forecasting task (Fig. 5d,f), the skyrmion-dominated reservoir (*H*_c_ = 15 mT) outperforms the ferromagnetic reservoir (*H*_c_ = 60 mT) in terms of the MSE. By contrast, the ferromagnetic reservoir can yield a better MSE than the skyrmion-dominated reservoir for the transformation task (Fig. 5e,g). See Supplementary Fig. 10 (Supplementary Note 8) for the full phase tunability of Co_8.5_Zn_8.5_Mn_3_ and FeGe. Although there is clear space for improving the MSE as well as making full use of the task-adaptive nature of this material system, this above-room-temperature demonstration shows no fundamental limit to using the task-adaptive concept in a wide variety of materials.

## Outlook

We have demonstrated the substantial benefits of introducing a phase-tunable approach, and hence task adaptability, to physical reservoir systems. A single physical reservoir may now be actively reconfigured on-demand for a strong performance across a broad range of tasks without the requirement for fabricating additional samples or using entirely different physical systems. This approach invites further development, such as online training and dynamic on-the-fly reservoir reconfiguration for incoming real-time datasets. Moreover, the phase-tunable approach demonstrated in our study can be transferable to a broad range of physical reservoirs, not only to magnetic materials that host chiral spin textures^47,49,50^ but also potentially to non-magnetic systems that have rich thermodynamical phase diagrams. This approach may also offer additional functionality for wave-based physical recurrent neural networks^32^. Experimental demonstration of on-demand reservoir reconfigurability brings physical reservoir computing closer to fully realizing its promise and helps to develop an alternative to software-based neural-network approaches powered by complementary metal–oxide–semiconductor technology software.

## Methods

### Details of experimental setup

#### Ferromagnetic resonance

For our experiments, a polished plate-shaped bulk Cu_2_OSeO_3_ crystal of dimensions 1.9, 1.4 and 0.3 mm (*x*, *y* and *z*, respectively) was placed on a coplanar waveguide with the (100) surface facing down. Here, the Cu_2_OSeO_3_ crystal was placed on a coplanar waveguide board, which sits on a copper cold finger of a closed-cycle helium cryostat. Here, we apply an external magnetic field *H* along the 〈100〉 crystallographic direction for the efficient generation of low-temperature skyrmions^40,41^. A VNA (ZNB40, Rohde & Schwarz) is employed to measure the spectral response of chiral magnetic crystals using ferromagnetic resonance (FMR) techniques. The microwave reflection coefficient *S*_11_ is recorded by the VNA as a function of the microwave frequency to characterize the spectral response for given magnetic fields and temperatures. For our measurements, we sweep the frequency, comprising 1,601 frequency points (*M*) at 0 dBm applied microwave power. Thus, a single raw spectral recording of *S*_11_ consists of 1,601 points, associated with the frequency dependence of the dynamic magnetic susceptibility *χ*_m_.

#### Field-cycling scheme

In standard field-cycling schemes without envelope modulation, a single field loop *N* is completed when *H* is increased and decreased between fixed field points, for example, defined by *H*_low_ (yellow), *H*_mid_ (red) and *H*_high_ (green) in Supplementary Fig. 1a, with different time steps of *t* as labelled. During the cycling process, the VNA records the corresponding spectrum for each magnetic field value to study the nucleation of metastable lattices such as low-temperature skyrmions^40,41^. This cycling scheme, however, lacks the ability to construct a time-series input function for reservoir computing.

We have, therefore, established the mapped field-cycling scheme to apply the field-cycling data-input protocol for physical reservoir computing. This technique, as shown in Supplementary Fig. 1b, modulates each of *H*_low_, *H*_mid_ and *H*_high_ for different *t* to generate a field-cycling-dependent input function *u*′(*N*). This makes it possible to incorporate arbitrary time-series signals *u*(*t*) in our scheme. For the mapping procedure, *u*(*t*) is normalized between −1 and 1 and is offset by a central cycling field value *H*_c_, where two additional copies (*H*_high_ and *H*_low_) are generated above and below *H*_mid_ using the cycling width *H*_range_. In this work, we accommodated two specific input sequences to suit different target applications: a chaotic oscillatory Mackey–Glass time-series signal^44^ (for forecasting) and a sine wave (for transformations). We construct the reservoir outputs using the FMR spectra measured at the lowest field point (yellow dots) within the cycles.

The mapped field-cycling scheme thus enables FMR frequency multiplexing. Frequency multiplexing is a technique commonly used to broadcast a single-dimensional input signal to multiple outputs. In our experiments, each field point is encoded as a series of frequencies applied to the magnetic system. By measuring the FMR response, multiple output signals at different frequencies can be separated and analysed in the spectral space to be used for computation.

A typical time to solve tasks is, in total, around two hours. The breakdown of this entire process is: (1) input field mapping as pre-processing (less than one minute), (2) inputting data as a magnetic field and recording the physical reservoir output via the VNA (two hours) and (3) training/testing the reservoirs (less than one minute). For the reservoir construction process, we use the VNA to acquire the frequency spectra, which take approximately one second for each spectrum. Changing the magnetic field dominates the measurement time, and the timescale is limited by this speed. All processing in this work, including reservoir computing, is conducted by a CPU (Ryzen 9 5900X, AMD).

### Details of reservoir computing protocols

#### Data processing

After completing a set of mapped field-cycling measurements, the spectral data are pre-processed before being added to the reservoir matrix *R*, as shown by the example in Supplementary Fig. 2a. Each spectrum undergoes the same processing method of a high-field (300 mT) background subtraction, a numerical lossless smoothing accommodated by the Savitzky–Golay filter^51^ and spectrum sampling at fixed intervals to obtain Δ*S*_11_. Data sampling is necessary to avoid an overfitting problem caused by too many data points during training (see Supplementary Note 2 and Supplementary Fig. 5 for more details). The sampling interval is determined by an automated search process that best produces the MSE of the test data.

#### Target generation

The transformation targets shown in the main text have been generated using the scipy.signal package^52^, where the input array is defined by 0.2π{1…*N*} for the ‘square’ waveform with a duty cycle of 0.5 and a ‘saw’ signal with a width of the rising ramp as 1. Note that the square target waveform has a very slight slope between the high and low values due to the finite sample rate.

For the forecasting tasks, Mackey–Glass, a chaotic time series derived from a nonlinear time-delayed differential equation, was employed. Its complex behaviour is commonly used as a benchmark for testing the performance of prediction algorithms. The signal is defined by: $\frac{{{{\rm{d}}}}x}{{{{\rm{d}}}}t}=\beta \frac{{x}_{d}}{1+{x}_{d}^{\,n}}-dx$, where *x* and *x*_d_ represent the value of the signal at time *t* and *t*-*d*, respectively. We have numerically generated the signal to exhibit a chaotic oscillatory behaviour with the following parameters: *β* = 0.2, *n* = 10, and *d* = 17.

#### Training and testing

For training and testing, *R* is subsequently separated into training and test datasets determined by a test-length factor *k*, in the ranges of [0, 1], as illustrated in Supplementary Fig. 2b. The training dataset is passed on to a variant of the linear regression algorithm, that is, ridge regression^53^, to calculate the optimal weights to reproduce the target dataset. Ridge regression is a common regression technique with a regularization term *α* for analysing multicollinear data. The weights are determined via $\left.\min(w)\right)\left | \right | \chi w-y \left | \right |_{2}^{2}+\alpha \left | \right |w\left | \right |_{2}^{2}$, where *w*, *χ* and *y* denote the ridge coefficients (weights), reservoir elements and the target value terms, respectively. Here, *α* helps to penalize large *w* values obtained during the fitting process to stabilize the model and prevent overfitting. We have used the scikit-learn package for this calculation^54^. The obtained weights are then applied to the unseen test dataset to evaluate the training performance and compared with the test target data.

In this study, *k* was fixed at 0.3, that is, using 70% of data for training and 30% for testing with *N* = 1,000 cycles. The dependence of *k* on the MSE values for the forecasting (transformation) task is shown in Supplementary Fig. 2c (2d). As observed in these plots, sufficient training data are necessary to improve the MSE for each case; in other words, *k* should be reasonably smaller than unity. We confirm that the choice of *k* does not substantially alter our analysis and conclusions drawn in this study.

#### Performance evaluation of reservoir computing

The MSE is a statistical measure that quantifies the difference between the predicted and true values by averaging the squared differences across data points. A lower MSE value indicates a better predictive performance for a given task. We calculate our MSE values using the mean_squared_error function of the sklearn.metrics package^54^, which evaluates: ${{{\rm{MSE}}}}(\,y,\hat{y})=\frac{1}{{n}_{{{{\rm{samples}}}}}}\mathop{\sum }\nolimits_{i = 0}^{{n}_{{{{\rm{samples}}}}}-1}{({\,y}_{i}-\hat{{y}_{i}})}^{2}$, where *y*_*i*_ and *ŷ*_*i*_ correspond to the target (true signal) and transformed/predicted values, respectively, and each consists of *n*_samples_ number of data points. Here *y*_*i*_ and *ŷ*_*i*_ is the *i*-th sample of *y* and *ŷ*.

### Correlation analysis

We determine the correlations for the MSE metric and the task-agnostic metrics using Spearman’s rank correlation coefficient^46^, which is a non-parametric measure (that is, it does not assume that the data follows a specific statistical distribution such as the normal distribution) of the strength and direction of association between two variables. It reflects the degree to which their rankings correlate, yielding values ranging from −1 to 1. The value of −1 indicates a perfect negative association (that is, where one variable increases, the other decreases). Conversely, the value of 1 implies a perfect positive association (where one variable increases, the other decreases).

Here we present correlation plots which we do not show in the main text. For this analysis, we normalize the MSE values as follows:

1 $${{{{\rm{MSE}}}}}^{{\prime} }=\frac{{\log }_{10}({{{\rm{MSE}}}})-\min ({\log }_{10}({{{\rm{MSE}}}}))}{\max ({\log }_{10}({{{\rm{MSE}}}}))-\min ({\log }_{10}({{{\rm{MSE}}}}))}.$$

Note that a log value of MSE was taken to minimize the correlation anomalies arising from a large range of MSE values, resulting in an incorrect representation of the dataset. This is equivalent to plotting the MSE values on a logarithmic scale.

## Online content

Any methods, additional references, Nature Portfolio reporting summaries, source data, extended data, supplementary information, acknowledgements, peer review information; details of author contributions and competing interests; and statements of data and code availability are available at 10.1038/s41563-023-01698-8.

### Supplementary information

Supplementary Information Supplementary Figs. 1–10 and Notes 1–8.

## Data Availability

The data presented in the main text and the Supplementary Information are available from the corresponding authors upon reasonable request.

## Code availability

The code used in this study is available from the corresponding author upon reasonable request.
