# Supplementary material for: Task-adaptive physical reservoir computing
Source: Nat Mater. 2023 Nov 13;23(1):79–87. doi: 10.1038/s41563-023-01698-8 (PMC10769874; doi:10.1038/s41563-023-01698-8)
Supplement: Supplementary file 1 — Supplementary Figs. 1–10 and Notes 1–8. [file 41563_2023_1698_MOESM1_ESM.pdf]

nature materials

Article https://doi.org/10.1038/s41563-023-01698-8

Task-adaptive physical reservoir computing

In the format provided by the
authors and unedited

Contents

Supplementary Note 1: Magnetic phases and tunability in Cu_2OSeO_3 **3**

Supplementary Note 2: Computational properties and physical characteristics **5**

Supplementary Note 3: Additional reservoir computing performance **7**

Supplementary Note 4: Details of reservoir metric calculations **9**

Supplementary Note 5: Additional reservoir metrics data **10**

Supplementary Note 6: Additional ac susceptibility data for $Co_{8.5}Zn_{8.5}Mn_3$ **11**

Supplementary Note 7: Task-adaptable physical reservoir computing on FeGe **11**

Supplementary Note 8: High-temperature task-adaptability **13**

References **14**

List of Figures

1 Details of field-cycling protocol . . . 2
2 Details of data processing . . . 3
3 Additional spinwave spectra data . . . 4
4 Summary of computational properties and physical characteristics . . . 5
5 Computational performance on the number of spectral points . . . 6
6 Additional reservoir computing performance data . . . 8
7 Additional reservoir performance and metrics data . . . 10
8 Additional ac susceptibility data for $Co_{8.5}Zn_{8.5}Mn_3$ crystal . . . 11
9 Summary of reservoir computing with FeGe . . . 12
10 Summary of task-adaptability on FeGe and $Co_{8.5}Zn_{8.5}Mn_3$ . . . 13

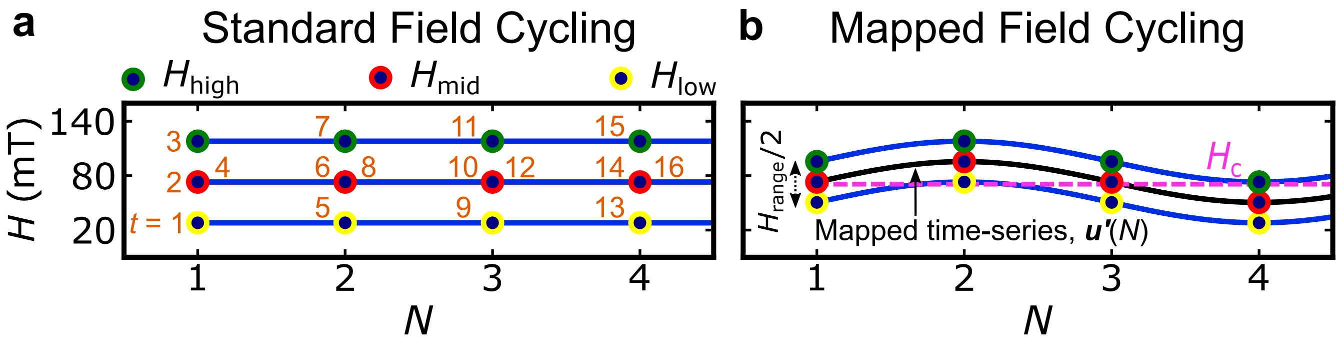

Supplementary Figure 1. Details of field-cycling protocol. a&b, Comparison of standard field-cycling (**a**) and mapped field-cycling schemes (**b**). Typical input scheme as a function of N for H_c = 73 mT and H_{range} = 90 mT. Here, t denotes the order of applied field values and measurement points. In standard cycling schemes, the lower and the upper field values are fixed, whereas, in mapped field-cycling, such field values are modulated by the input function at a central field value H_c with a fixed separation between the upper (H_{high}) and the lower (H_{low}) cycling fields, described by $H_{range}/2$.

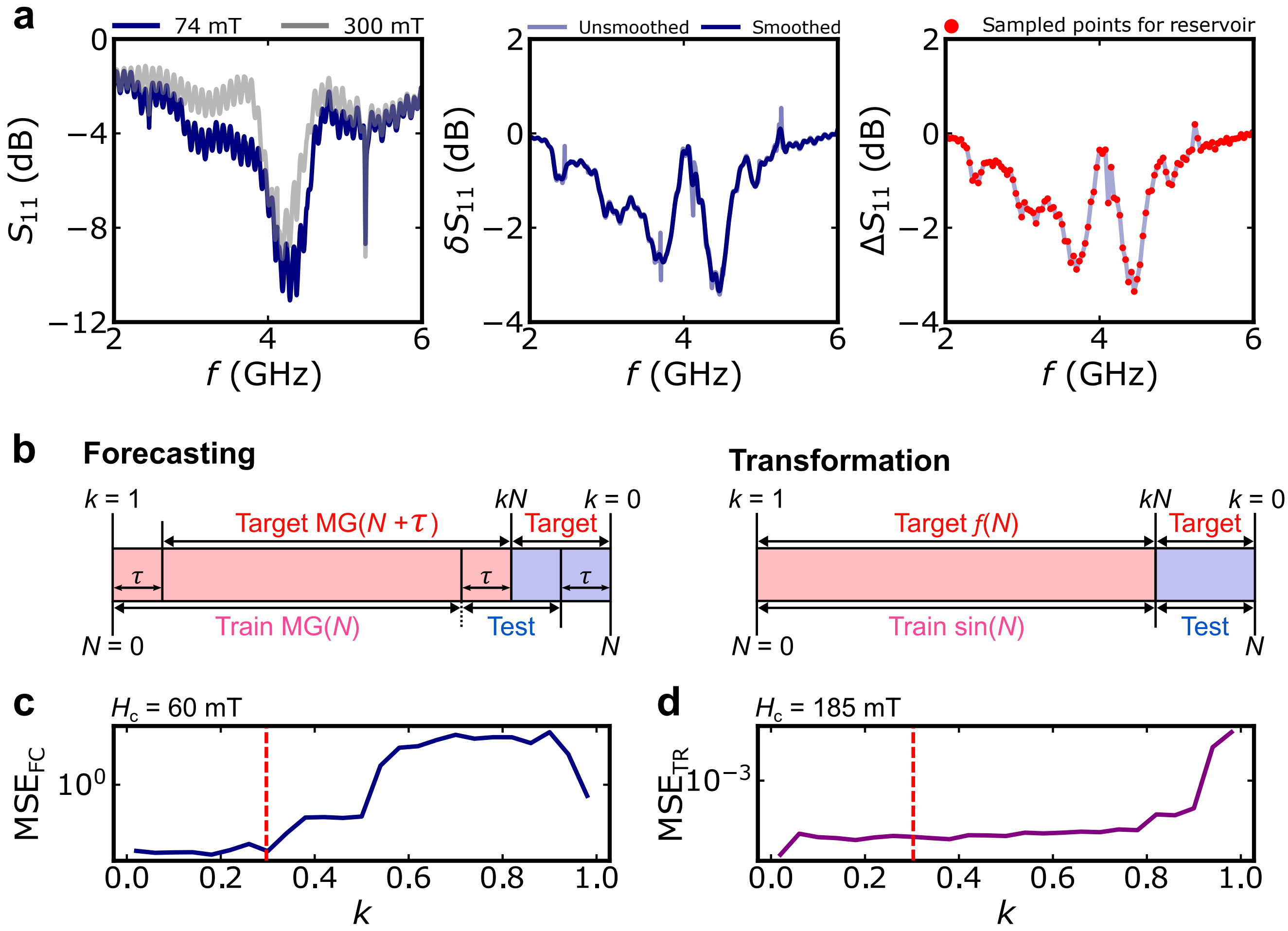

Supplementary Figure 2. Details of data processing. **a**, Definition of ΔS_{11}; a smoothed spectra (δS_{11}) is sampled at fixed intervals after subtracting high-field spectra from the raw signal (S_{11}). **b**, Illustration of training and testing dataset. For forecasting, the weights are calculated on the training readouts of the reservoir constructed using a Mackey-Glass input MG(N) to predict the target MG($N+\tau$), defined at τ future steps from the original. For transformations, the reservoir is created using a sinewave input sin(N), where the weights are calculated on the training data to best reproduce a target function $f(N)$ that is different to a sinewave. Here, k is a constant multiplier between [0, 1] that determines the length of the test set data. **c&d**, MSEs of forecasting and transformations as a function of k. The red dotted line highlights the chosen value of k used in this study (0.3).

Supplementary Note 1: Magnetic phases and tunability in Cu_2OSeO_3

Cu_2OSeO_3 is one of chiral magnets, having a noncentrosymmetric cubic lattice belonging to the $P2_13$ space group where the competition of symmetric/anti-symmetric exchange, magnetic-dipole and Zeeman interactions provides different magnetic phases[1,2]. As shown in Fig. 2a in the main manuscript, it possesses four different magnetic phases in the temperature-magnetic-field phase diagram. For small magnetic fields, spins in Cu^{2+} ions point as spiral rotation within a specific plane, hence having the corresponding modulation vector. This is called the helical phase. When the magnetic field is increased, there is a finite spin component along the field direction for each Cu^{2+} ion, forming the conical state. Finally, when the magnetic field is further increased, the spiral component is completely lost, having the three-up/one-down spin configuration. This is called the ferrimagnetic state[1]. Furthermore, skyrmion phases tend to form between the helical and conical phases at high-temperature pockets closely below the Curie temperature T_c[1,2]. However, a distinctive thermodynamically metastable skyrmions can also be realised at lower temperatures[3,4]. Their population can be controlled by the number of field-cycling[5,6], making them an adequate candidate to perform reservoir computing. From a detailed previous study[6], the sample is anticipated to host this phase at temperatures below $\sim$25 K and magnetic fields between $25 < H < 120$ mT.

Figure 3 summarises the spectral evolution by a sinewave input signal for different H_c. These plots use the same dataset that generate colour plots of Fig. 2e in the main manuscript. For H_c = 35 mT, the magnet is predominated by the helical phase

(Fig. 3a) and its resonant modes around 4 GHz are assigned as $\pm Q$ modes in the magnetic phase[5–7]. However, increasing H_c to 60 mT and 70 mT (Figs. 3b&c), clear signatures of skyrmions are seen (between 2 - 3 GHz), where their FMR positions are subtly modulated by the input signal. At H_c = 98 mT (Fig. 3d), approximately half of the input signal is cycled within the skyrmion phase and the other half in the conical phase. This results in a hybridised state where both skyrmion and conical modes share the lattice. Furthermore, when field-cycling predominantly occurs outside of the skyrmion phase, i.e., H_c = 185 mT (Fig. 3e), the conical reservoir excellently encodes the input signal as shown by their FMR positions, comparable to the amplitude of the input fields as depicted in Fig. 3. This mode yields a high nonlinearity (NL) and complexity (CP), leading to an outstanding performance of transformation tasks.

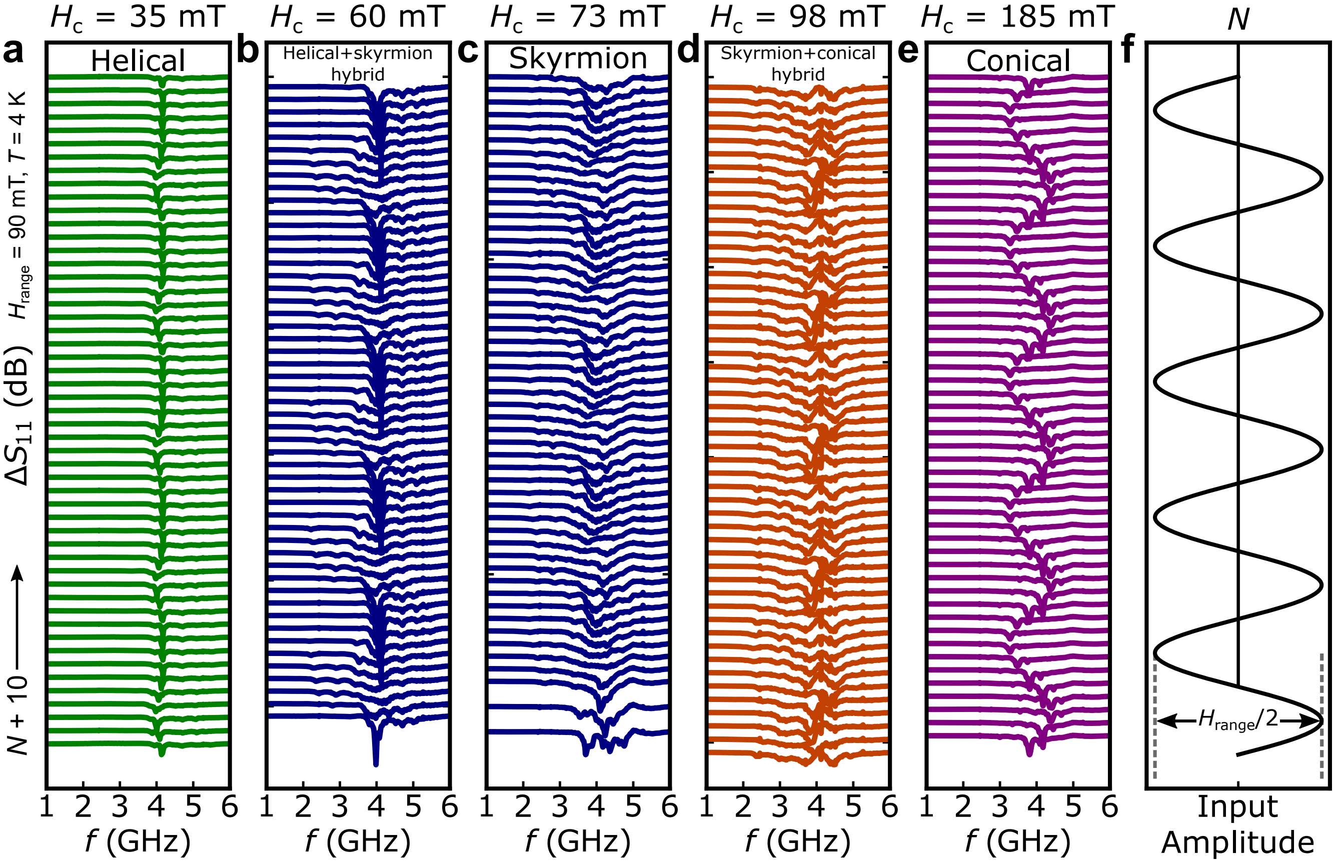

Supplementary Figure 3. Additional spinwave spectra data. Evolution of spectra in Cu_2OSeO_3 as a function of microwave frequency f for increasing values of N for a sinewave input signal at different values of H_c with H_{range} = 90 mT at a constant temperature T = 4 K. Each spectrum represents the completion of 10 field-cycles from its previous. By tuning H_c, the reservoir can be constructed with different dominant magnetic phase-spaces: **a**, helical, **b**, helical + skyrmion hybrid, **c**, skyrmion, **d**, hybrid (skyrmion + conical), and **e**, conical. **f**, Applied input amplitude as a function of N used to construct reservoirs in **a**-**e**.

Supplementary Note 2: Computational properties and physical characteristics

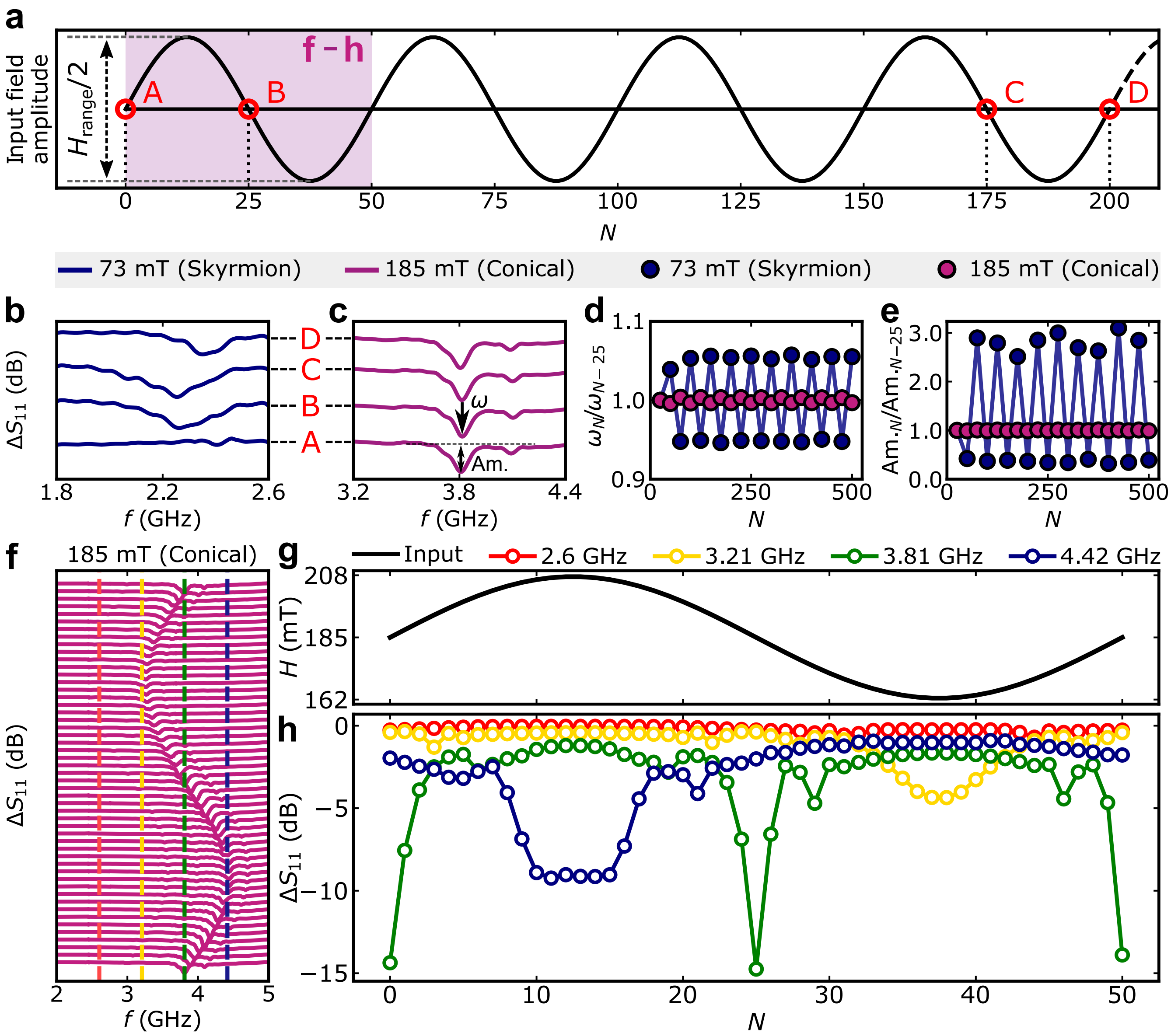

Supplementary Figure 4. **Summary of computational properties and physical characteristics.** Spectral evolution and feature analysis of physical reservoirs using Cu_2OSeO_3. **a**, Illustration of the MFC scheme for defining input amplitudes, with points marked “A" to “D" representing input fields analyzed in **b**-**e**. The purple shaded region highlights a single period of sinewave used for analysis in **f**-**h**. **b**&**c**, Individual spectra highlighting counterclockwise skyrmion and conical modes, respectively. **d**&**e**, Relative changes in peak frequency (ω_N/ω_{N-25}) and peak amplitude ($\mathrm{Am.}_N/\mathrm{Am.}_{N-25}$), measured at each node on the sinewave input, as an evolution of N. Dots and lines in blue (purple) represent the skyrmion (conical) mode. **f**, Cycling-evolution of spectra at conical phase ($H_c = 185$ mT) as a function of frequency. The dashed vertical lines depict the frequencies evaluated for **h**. **g**, First period of sinewave input fields as a function of N. **h**, Spectral amplitudes as a function of cycling number N at various fixed frequency channels.

Here we further analyse results in Fig. 3 to describe our interpretation of why individual magnetic phases perform differently and are suited for the specific tasks we present. We show in Fig. 4, our further analysis of each spectrum recorded at the exact field strengths (73 and 185 mT) but at different points. Figure 4a summarises our sine input function field series with cycle number N and specifies Points A - D all having the same field value but different N, i.e. A ($N = 0$), B ($N = 25$), C ($N = 175$) and D ($N = 200$), respectively. For the spectral evolution at the centre field of 73 mT targeting at the skyrmion

modes in Cu_2OSeO_3 (Fig. 4b), all of the frequency spectra (A - D) are dissimilar to each other, although they are measured at the exact same magnetic field value. For example, the spectra at A (C) and B (D) are separated by the half period of the sine input function and this field history is imprinted as their magnetic properties, i.e., its absorption properties ΔS_{11}. This clearly shows short-term memory-capacity (MC) in the skyrmion phase: *i.e.* $x(N) = f(\ldots, u'(N-1), u'(N), x(0))$ where $x(N)$ is the state vector of a reservoir at the field-cycling point N and $u'(N)$ is the input function at N.

Moreover, Points B and C are connected by periodic translation, and their spectra have the same peak position but different heights. This is due to the cycling-number-dependent meta-stable skyrmion population – the more we cycle, the more we nucleate the skyrmions[5,6]. This intrinsic material property generates additional (long-term) memory in the reservoir, being able to perform superbly for tasks requiring strong MC, such as future prediction. High performance (MSE) of the skyrmion phase on our benchmarking of predicting the nonlinear Mackey-Glass time series function, correlated with high values of MC, is robust evidence of this claim. In this task, the relationship between input data excitation and target output response (i.e. prediction value) is constantly evolving throughout the Mackey-Glass time series due to its chaotic nature. In order to reliably predict these ever-changing targets, the state of the reservoir must hold enough information about past states (the short-term memory) to accurately discriminate the precise nature of the chaotic behaviour at the current position in the time series.

This unique memory property is absent for reservoirs dominated by the other two magnetic phases as observed in Fig. 4c for the conical reservoir where spectra for Points A - D are all identical, therefore history-independent. To quantitatively discuss the difference in the reservoir memory property, we performed a single Lorentzian fit[1] to each spectrum shown in Figs. 4b&c for extracting their peak position (ω_N) and amplitude (Am.$_N$). We plot the ratio of ω_N/ω_{N-25} and Am.$_N$/Am.$_{N-25}$ for both skyrmion and conical reservoirs in Figs. 4d&e. Both plots clearly support the strong memory property in the skyrmion reservoir.

Instead, the conical reservoir is equipped with high NL and CP, yielding strong performance in the transformation tasks that require these properties. To feature the strong NL/CP property of the conical mode, we plot individual spectra for one sine input function period (Fig. 4f) together with its spectral value evolution with N in Fig. 4h. The main peak of the conical phase moves in a similar manner as the input magnetic field, as demonstrated in Fig. 4f. Individual frequency values plotted in Fig. 4h have drastic changes in amplitude, distinctly different from the sine input function plotted above (Fig. 4g). Each frequency point has unique evolution offering rich nonlinear responses as a whole. This large set of diverse responses to the input function empowers the reservoirs in performing signal transformation tasks, as numerically quantified in our metrics, i.e. NL and CP.

Furthermore, the size of reservoirs, i.e. the number of spectral points used for developing our reservoir, is found to be critical in our case. As shown in Fig. 5, MSE is greatly improved when the spectral point is increased, except for the future prediction task with the conical reservoir where no memory properties are expected, suggesting that adding more spectral points with no memory does not improve computing performance significantly. Since each spectral point behaves differently by the input function of the magnetic field due to nonlinearity, this high-dimension mapping is very efficient, producing an excellent performance for the benchmarking tasks used in our study.

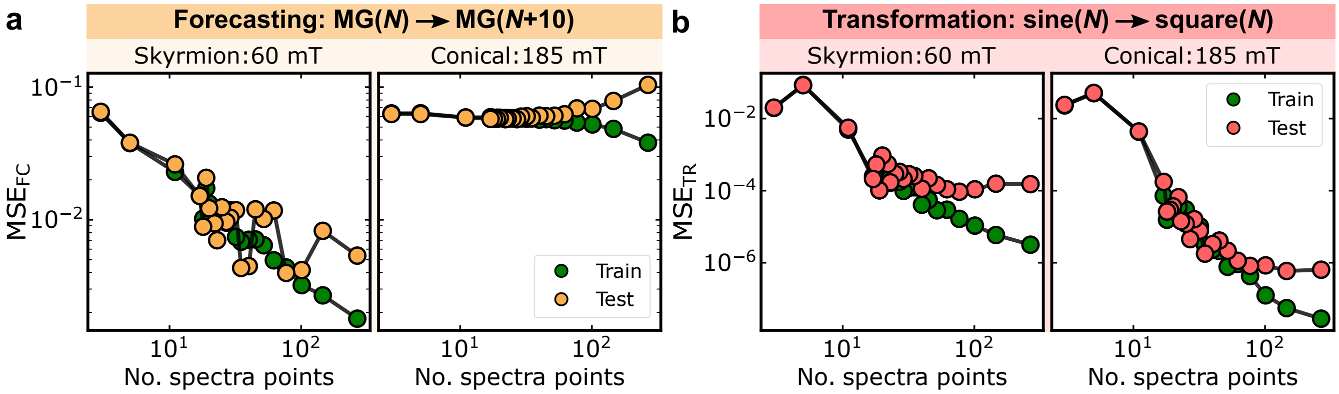

Supplementary Figure 5. **Computational performance on the number of spectral points. a&b**, Spectral size-dependence of MSE values for forecasting (MG($N+10$); **a**) and nonlinear transformation (sine to square; **b**). The left column depicts a skyrmion-dominated region (60 mT), while the right column represents a conical mode-dominated region (185 mT). Green dots denote the training data and yellow (red) dots represent test results for the forecasting (transformation) tasks.

[1] A curve-fitting technique commonly used to extract physical quantities from the FMR response. Defined by: $\Delta S_{11} \propto (\mathrm{Am.}\Delta\omega)/((f-\omega)^2 + \Delta\omega^2 + \mathrm{C})$, where Am., $\Delta\omega$, f, ω, and C, represents the peak amplitude, linewidth, frequency, peak frequency position, and offset constant, respectively.

Supplementary Note 3: Additional reservoir computing performance

Next, we present additional reservoir computation results on a broader range of tasks to further support our claims in the main manuscript. Figure 6a shows the training and testing performance for nonlinear transformation from a sinewave input signal to a range of different target waveforms. The target data for the triangle and Gaussian signals are generated using the `scipy.signal` package[8], similar to the square target waveform. Note that a triangle signal is a form of a saw signal with a symmetric width of 0.5. A periodic Gaussian pulse is generated by concatenating Gaussian signals with a standard deviation of 5. A sine-squared target is constructed by squaring a numerically evaluated sinewave with the same input array used for the square. A cosine waveform has been generated in a similar fashion. A hysteretic signal is a form of a second-order nonlinear equation where the output is dependent on its previous value[9]. A combined signal is arbitrarily generated by multiplying a square waveform by all signals shown in the figure (square$(N) \times ... \times$ hysteretic(N)). In all cases, the transformation MSE ($\mathrm{MSE_{TR}}$) values are within the magnitudes of 10^{-3} or below, showing excellent transformation performance across a diverse range of target signals.

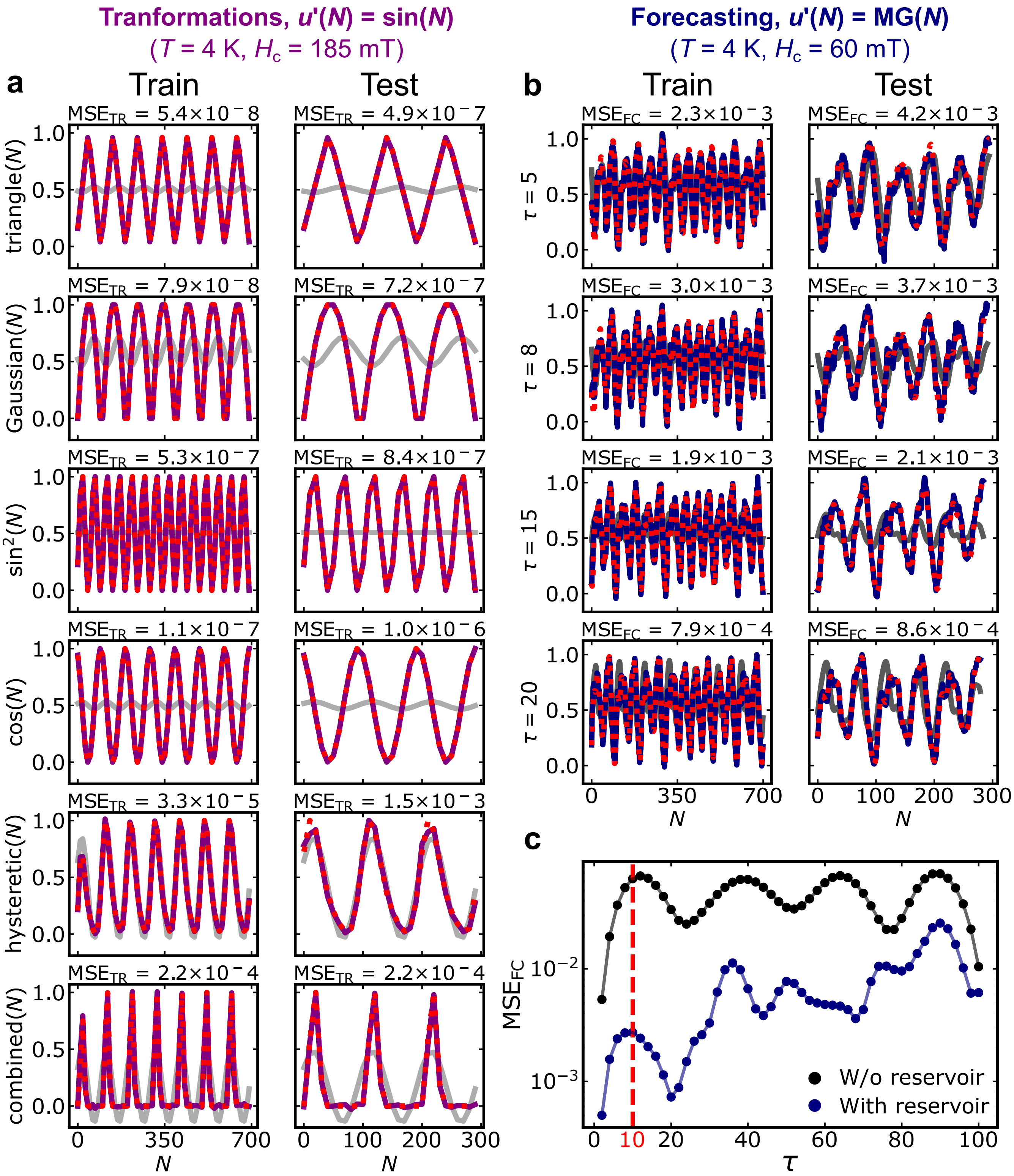

Supplementary Figure 6. Additional reservoir computing performance data. a&b, Training and testing transformations (**a**) of a sinewave input signal into various target functions with H_c = 185 mT (conical), and forecasting (**b**) of a Mackey-Glass signal for different future steps τ with H_c = 60 mT (skyrmion). Both tasks use H_{range} = 90 mT at T = 4 K. The grey lines are transformation/prediction computation without using the reservoir element. **c**, Forecasting MSE as a function of τ using the same conditions as **b** with and without reservoir. The prediction performance without the reservoir shows an intrinsic periodicity of $\tau \approx 22$, where a red line is drawn at $\tau = 10$, representing the presented and the analysed value of τ in the main text.

Figure 6b shows the training and testing data for a forecasting task of the chaotic Mackey-Glass input signal MG(N) for a range of different future steps τ. As shown in Fig. 6c, although the Mackey-Glass signal is chaotic, it is also quasi-periodic with an approximate period of $\tau \approx 22$ (see the black dots). Thus prediction performance ($\mathrm{MSE_{FC}}$) also varies periodically, as predicting a full period ahead requires far less modification of the input signal to accurately recreate the target signal, as evidenced by the observed periodic relation between $\mathrm{MSE_{FC}}$ and τ. Throughout this study, we have consistently chosen $\tau = 10$ where the computation of forecasting is nontrivial within the first quasi-period in order to best evaluate the computational power of our physical reservoir system.

Supplementary Note 4: Details of reservoir metric calculations

We evaluate our reservoir metrics (NL, MC, and CP) based on prior works described in refs.[10–13]. The underlying scope of these metrics is to quantify how the reservoir states $\chi(N)$ responds to a given input signal, $\boldsymbol{u'}(N)$. While a random input dataset is often used for such metric calculations, here, we perform metric calculations on the output data from the chaotic Mackey-Glass dataset as the input, as employed in prior studies[9]. We use $\boldsymbol{u'}(N)$ = MG(N) at H_{range} = 90 mT for different values of H_{c} and temperatures to measure the metrics in this work. Before the evaluation, reservoir outputs which are unresponsive to the input signal (i.e. FMR frequency channels in 'dead' frequency ranges where no FMR resonance modes are present) and potentially introduce artefacts in reservoir metric calculations are removed. This is achieved by ranking the FMR frequency output channels by their range (maximum value minus minimum value of that frequency channel over the entire set of N) and selecting the top 30% of FMR frequency channels with the highest range to perform our reservoir metric calculations. Channels with low range are effectively noise dominated 'dead' regions of the spectra and do not contribute meaningful reservoir metric information.

NL returns a score between [0, 1], where the value of 0 (1) represents a completely linear (nonlinear) system[13]. This metric evaluates the ability of the reservoir to predict the true readout χ_{m} (FMR frequency channels) when shown up to 8 previous inputs, as shown in Eq. S1.

$$\hat{\chi}_m(N) = \sum_{i=0}^{8} w_i u'(N-i) \tag{S1}$$

Here, the weights w are calculated using linear regression with 750 cycles for training. Subsequently, w is applied to the unseen 250 cycles to obtain the prediction $\hat{\chi}_{\mathrm{m}}$, which is compared with corresponding χ_{m} through R^2 coefficient of determination (see Eq. 9 in Ref.[13] for details). This process is repeated for all values of FMR frequency channels (m = {1..480}), where NL is evaluated as:

$$\mathrm{NL} = 1 - \mathrm{AVERAGE}(\mathrm{R}^2[\hat{\chi}_{\mathrm{m}}, \chi_{\mathrm{m}}]) \tag{S2}$$

MC determines the reservoir's capacity to remember previous information about its inputs, i.e. how much past input information is present in the current readout datapoint[10,13]. The metric evaluates the performance of the current state of the reservoir (χ_m) in predicting up to its last 8 input states, as described in Eq. S3.

$$\hat{u}(N-i) = \sum_{m=1}^{480} w_m \chi_m(N) \tag{S3}$$

Here, we obtain w similar to the NL evaluation. However, we use the readout (χ_m) to predict the states of the previous input signals. This prediction is subsequently compared with the true state of the input via R^2 metric, where MC is evaluated (Eq. S4).

$$\mathrm{MC} = \sum_{i=1}^{8} \mathrm{R}^2 \left[\hat{u}(N-i), u(N-i)\right] \tag{S4}$$

A high MC score indicates that the reservoir retains a substantial amount of past input data in its current spectral information over a more extended period of past inputs.

CP determines the effective latent space of the reservoir, i.e., the amount of meaningful information encoded in the spectra[13]. Here, only the readouts of the reservoir are considered for the calculations. We first prepare the readouts into two square matrices, each of dimensions 480×480 to calculate the effective rank of the individual matrices[11,13], measuring the exponent of a Shannon entropy of normalised singular vector values evaluated using a singular value decomposition technique[14]. Subsequently, the average of the two effective ranks gives the CP score. Higher CP values indicate that the system is more perceptive to salient features in the input data.

In software reservoir computing, additional hyperparameter metrics including the spectral radius may be directly calculated via the matrix of internal reservoir weights (i.e. the fixed, randomised internal structure of the reservoir itself as opposed to the

task-specific training weights produced via linear regression). While this is not possible in physical neuromorphic computing systems, both as the internal reservoir structure shifts dynamically in response to input stimuli and as the internal reservoir structure is extremely challenging to fully quantify, the higher-level metrics (MC/NL/CP) which may be more readily assessed from the reservoir response have been shown to be strongly correlated with the internal reservoir hyperparameters. A large spectral radius correlates with strong nonlinearity, and a small spectral radius correlates with strong memory[15,16]. Hence, the ability of our phase-tunable approach to reconfigure MC, NL and CP metrics can be seen as evidence that our methodology is capable of dynamically reconfiguring reservoir hyperparameters such as the spectral radius and accordingly the internal reservoir connectivity and structure.

Supplementary Note 5: Additional reservoir metrics data

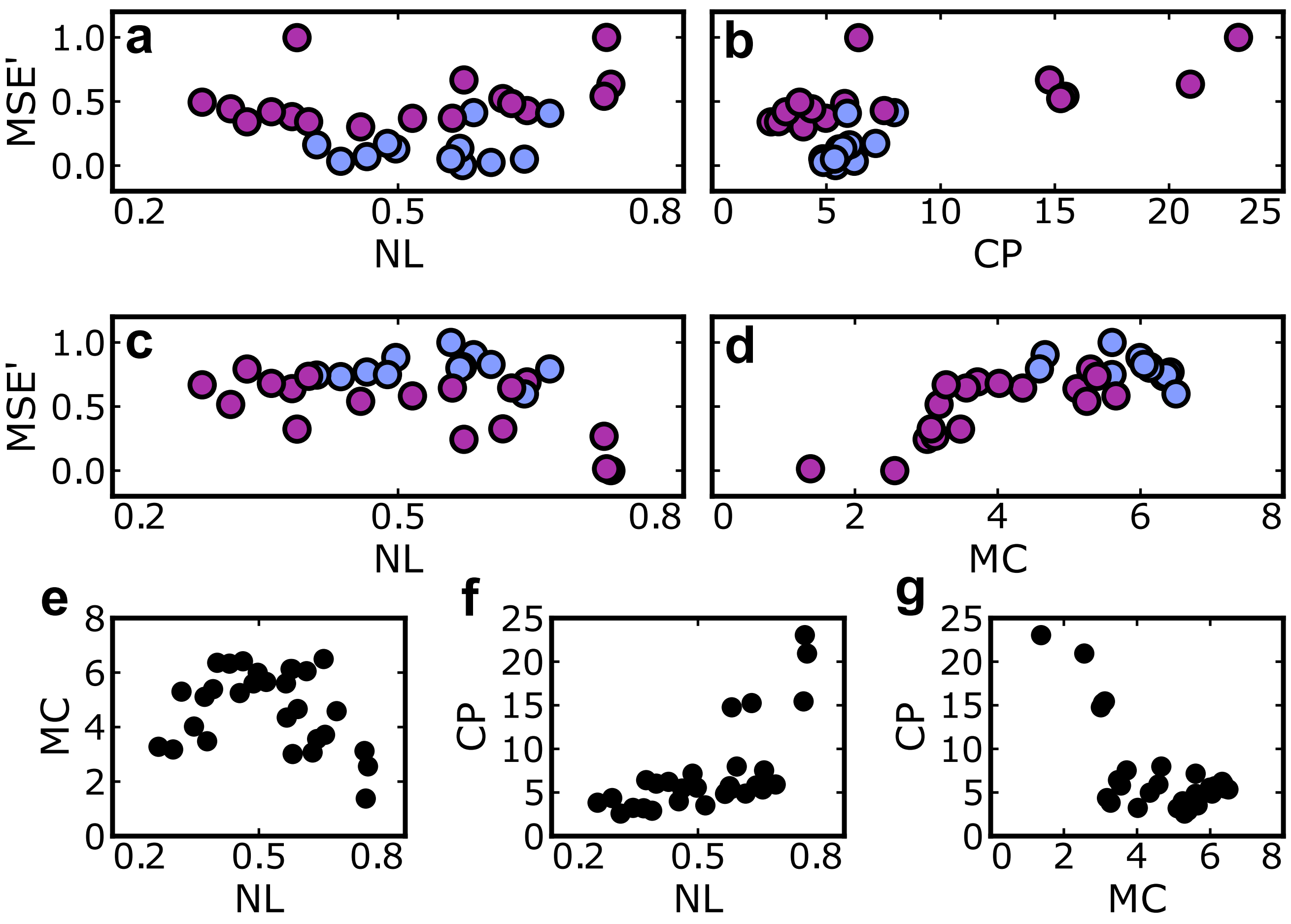

Supplementary Figure 7. Additional reservoir performance and metrics data. a&b, Forecasting performance as a function of NL (**a**) and CP (**b**). **c&d**, Transformation performance as a function of NL (**c**) and MC (**d**). **e**, MC as a function of NL. **f&g**, CP as a function of NL (**f**) and MC (**g**).

Figures 7a and b respectively show the MSE'_{FC} as a function of NL and CP. MSE'_{FC} and NL have a weak correlation of 0.14, whereas CP shows a positive correlation of 0.57. MSE'_{FC} is minimised when skyrmions (blue dots) are present. Similarly, Figs. 7c and d respectively show the transformation performance as a function of NL and MC. MSE'_{TR} negatively correlates with NL (-0.36) and has a strong (positive) correlation with the MC (0.77). In Figs. 7e-g, we show MC against NL (e), CP against NL (f) and CP against MC (g). In Fig. 7e, MC is shown as a function of NL, with a relatively low correlation (-0.27) and bell-curve like shape. Fig. 7f shows the CP as a function of NL, well positively correlated (0.67) with the highest scoring NL points also exhibiting high CP. Finally, Fig. 7g shows the CP as a function of MC, which has a pronounced negative correlation (-0.68).

Supplementary Note 6: Additional ac susceptibility data for $Co_{8.5}Zn_{8.5}Mn_3$

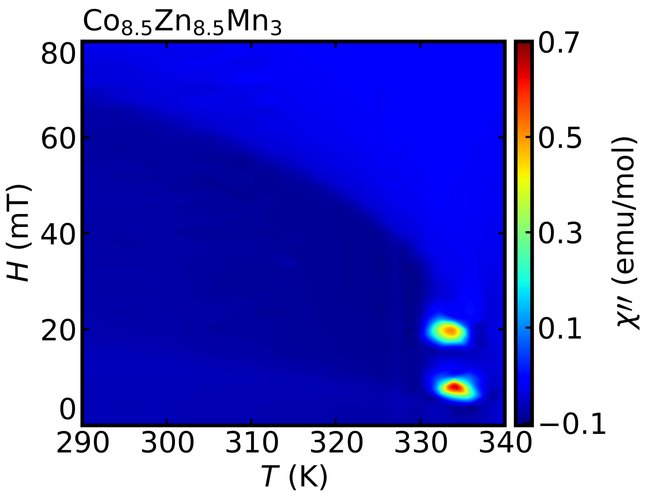

Supplementary Figure 8. Additional ac susceptibility data for $Co_{8.5}Zn_{8.5}Mn_3$ crystal. A 2D plot of the imaginary-part of ac susceptibility (χ") of the $Co_{8.5}Zn_{8.5}Mn_3$ crystal.

Magnetic susceptibility measurements are sensitive to magnetic phase changes. We performed ac susceptibility experiments for $Co_{8.5}Zn_{8.5}Mn_3$, yielding the real (χ'; see Fig. 5a in main text) and imaginary (χ'') components. Figure 8 shows χ'' of $Co_{8.5}Zn_{8.5}Mn_3$ with clear bright-regions in $330 < T < 340$ and $H < 25$, highlighting the presence of the skyrmion phase due to a slow relaxation process observed around the skyrmion phase[17].

Supplementary Note 7: Task-adaptable physical reservoir computing on FeGe

Similar to Cu_2OSeO_3 and $Co_{8.5}Zn_{8.5}Mn_3$ presented in this study, the chiral-lattice of FeGe also hosts a rich magnetic phase diagram, including skyrmions at near room temperature[18–20]. The following section summarises the phase-tunable approach using the FeGe sample at $T = 283$ K with 5 mT cycling width. In Figs. 9a&b, we present the magnetic resonance spectra during field-cycling for Mackey-Glass and sine input functions, which were used to perform future prediction of MG($N+5$) and transformation (sine to triangle) tasks shown in Figs. 9c-f. Evidently, the spectra strongly depend on the choice of H_c, highlighting the phase-tunability of physical reservoirs in this material system. In particular, for the forecasting tasks (Figs. 9c&d), the skyrmion-dominated reservoir (H_c = 31 mT) surpasses the conical reservoir (H_c = 66 mT) in terms of MSE score (skyrmion: 2.5×10^{-2} vs conical: 3.4×10^{-2}). However, for the transformation task, the conical reservoir achieves a better MSE than the skyrmion-dominated reservoir (skyrmion: 4.6×10^{-3} vs 2.6×10^{-3}). Providing further evidence of phase-tunability of achieving task-adaptability.

Reservoir: FeGe

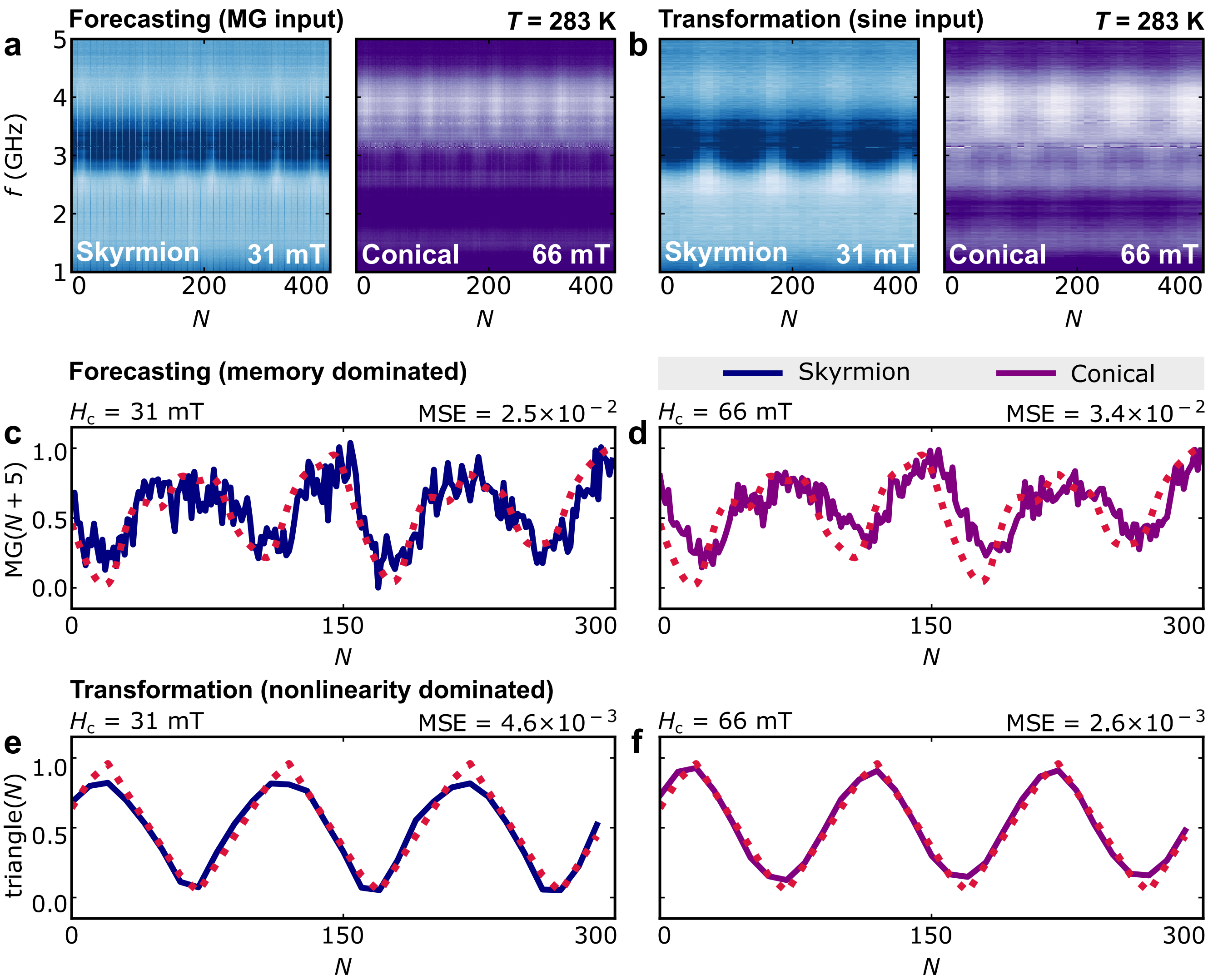

Supplementary Figure 9. Summary of reservoir computing with FeGe. Task-adaptive physical reservoir computing using FeGe at T = 283 K. **a**, 2D plots of spin dynamics spectra measured as an evolution of N for a Mackey-Glass and sine input functions at different values of H_c (31 and 66 mT). **b&c**, Reservoir computing performance for predicting the MG function with 5 future steps and transforming a sine input signal to a triangle output, respectively. The dotted curves/lines represent the target function, while the solid curves/lines demonstrate the success of our task-adaptive physical reservoir computing approach.

Supplementary Note 8: High-temperature task-adaptability

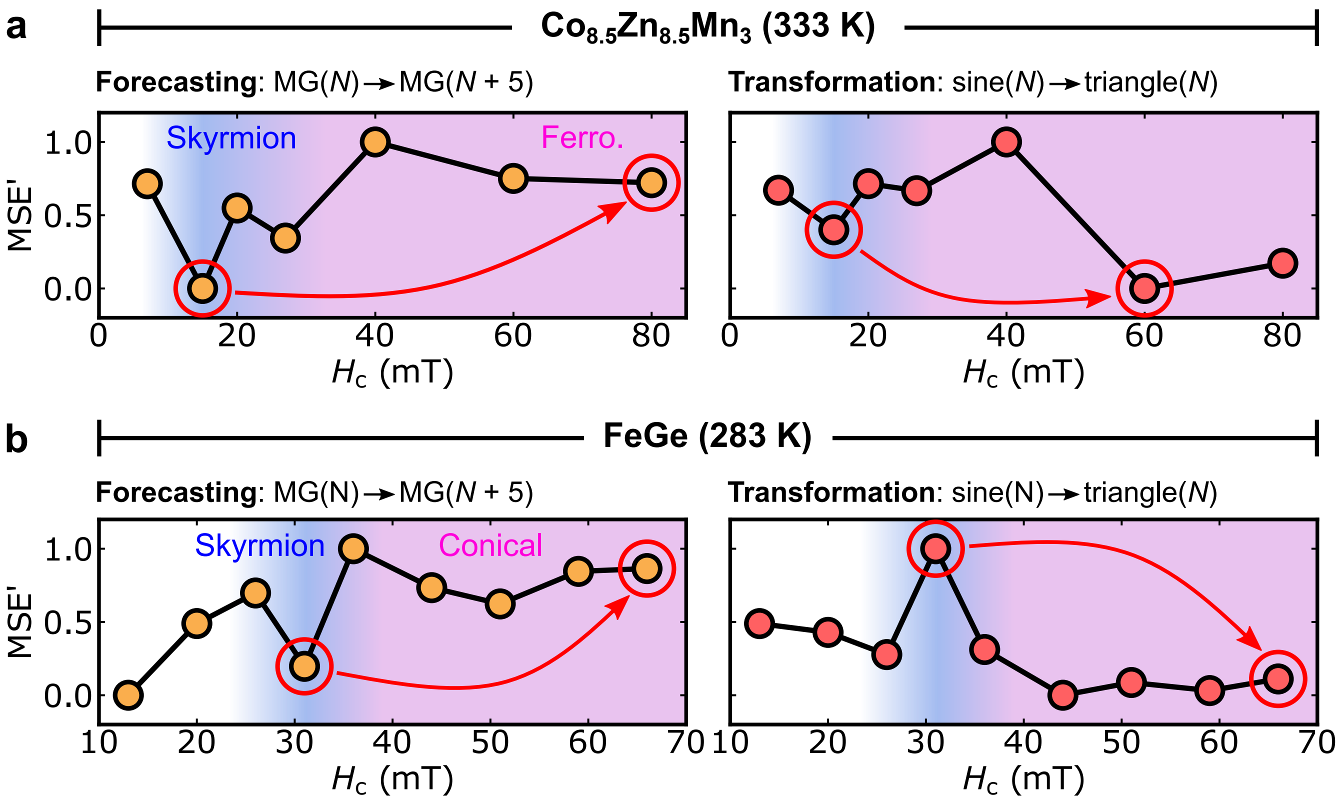

Supplementary Figure 10. Summary of task-adaptability on FeGe and $Co_{8.5}Zn_{8.5}Mn_3$. a&b, Comparison of task-adaptive physical reservoir computing using different materials and temperatures. Performance of forecasting a MG signal of five future steps and transforming a sine input signal to a triangle output function for $Co_{8.5}Zn_{8.5}Mn_3$ at T = 333 K (**a**) and FeGe at T = 283 K (**b**) as an evolution of H_c. Blue and purple backgrounds denote the skyrmion and ferromagnetic/conical regions.

In Fig. 10, we demonstrate the phase-tunability of chiral magnets including $Co_{8.5}Zn_{8.5}Mn_3$ and FeGe near room temperature for two distinct tasks: forecasting and transformation. Similarly to Fig. 4b in the main text for Cu_2OSeO_3 at 4 K, the task prediction performance is plotted against H_c. For forecasting tasks with $Co_{8.5}Zn_{8.5}Mn_3$ at 333 K (Fig. 10a), the skyrmion phase exhibits the best performance at H_c = 15 mT, which gradually decreases as the system transitions through the conical phase and into the ferromagnetic state. Conversely, for transformation tasks, the performance improves when moving from the skyrmion phase to the ferromagnetic phase (e.g., H_c = 15 to 60 mT), highlighting the ability of the system to transform a sine input function into a triangular wave output. The same behaviour maintains persistence for the FeGe sample at 283 K (Fig. 10b), i.e., the forecasting is best at the skyrmion phase (H_c = 31 mT) and decrease with increasing H_c, and vice versa for transformation tasks. These further support that the task-adaptive reservoir computing concept can be transferable to a wide range of different materials.

References

1. Seki, S., Yu, X. Z., Ishiwata, S. & Tokura, Y. Observation of skyrmions in a multiferroic material. *Science* **336**, 198–201, DOI: 10.1126/science.1214143 (2012).

2. Onose, Y., Okamura, Y., Seki, S., Ishiwata, S. & Tokura, Y. Observation of magnetic excitations of skyrmion crystal in a helimagnetic insulator cu$_2$oseo$_3$. *Phys. Rev. Lett.* **109**, 037603, DOI: 10.1103/PhysRevLett.109.037603 (2012).

3. Chacon, A. *et al.* Observation of two independent skyrmion phases in a chiral magnetic material. *Nat. Phys.* **14**, 936–941, DOI: 10.1038/s41567-018-0184-y (2018).

4. Halder, M. *et al.* Thermodynamic evidence of a second skyrmion lattice phase and tilted conical phase in cu$_2$oseo$_3$. *Phys. Rev. B* **98**, 144429, DOI: 10.1103/PhysRevB.98.144429 (2018).

5. Aqeel, A. *et al.* Microwave spectroscopy of the low-temperature skyrmion state in cu$_2$oseo$_3$. *Phys. Rev. Lett.* **126**, 017202, DOI: 10.1103/PhysRevLett.126.017202 (2021).

6. Lee, O. *et al.* Tunable gigahertz dynamics of low-temperature skyrmion lattice in a chiral magnet. *J. Physics: Condens. Matter* **34**, 095801, DOI: 10.1088/1361-648x/ac3e1c (2021).

7. Garst, M., Waizner, J. & Grundler, D. Collective spin excitations of helices and magnetic skyrmions: review and perspectives of magnonics in non-centrosymmetric magnets. *J. Phys. D: Appl. Phys.* **50**, 293002, DOI: 10.1088/1361-6463/aa7573 (2017).

8. Virtanen, P. *et al.* SciPy 1.0: Fundamental Algorithms for Scientific Computing in Python. *Nat. Methods* **17**, 261–272, DOI: 10.1038/s41592-019-0686-2 (2020).

9. Gartside, J. C. *et al.* Reconfigurable training and reservoir computing in an artificial spin-vortex ice via spin-wave fingerprinting. *Nat. Nanotechnol.* **17**, 460–469, DOI: 10.1038/s41565-022-01091-7 (2022).

10. Jaeger, H. *et al.* *Short term memory in echo state networks*, vol. 5 (GMD-Forschungszentrum Informationstechnik Bremen, Germany, 2001).

11. Roy, O. & Vetterli, M. The effective rank: A measure of effective dimensionality. *Eur. Signal Process. Conf. (EUSIPCO)* 606–610 (2007). http://infoscience.epfl.ch/record/110188.

12. Dambre, J., Verstraeten, D., Schrauwen, B. & Massar, S. Information processing capacity of dynamical systems. *Sci. Reports* **2**, DOI: 10.1038/srep00514 (2012).

13. Love, J., Mulkers, J., Bourianoff, G., Leliaert, J. & Everschor-Sitte, K. Task agnostic metrics for reservoir computing (2021). https://doi.org/10.48550/arXiv.2108.01512.

14. Shannon, C. E. A mathematical theory of communication. *Bell Syst. Tech. J.* **27**, 379–423, DOI: https://doi.org/10.1002/j.1538-7305.1948.tb01338.x (1948).

15. Butcher, J., Verstraeten, D., Schrauwen, B., Day, C. & Haycock, P. Reservoir computing and extreme learning machines for non-linear time-series data analysis. *Neural Networks* **38**, 76–89, DOI: https://doi.org/10.1016/j.neunet.2012.11.011 (2013).

16. Jaeger, H. *Tutorial on Training Recurrent Neural Networks, Covering BPPT, RTRL, EKF and the "echo State Network" Approach*. GMD-Report (GMD-Forschungszentrum Informationstechnik, 2002).

17. Qian, F. *et al.* Phase diagram and magnetic relaxation phenomena in cu$_2$oseo$_3$. *Phys. Rev. B* **94**, 064418, DOI: 10.1103/PhysRevB.94.064418 (2016).

18. Yu, X. Z. *et al.* Near room-temperature formation of a skyrmion crystal in thin-films of the helimagnet fege. *Nat. Mater.* **10**, 106–109, DOI: 10.1038/nmat2916 (2011).

19. Porter, N. A., Gartside, J. C. & Marrows, C. H. Scattering mechanisms in textured fege thin films: Magnetoresistance and the anomalous hall effect. *Phys. Rev. B* **90**, 024403, DOI: 10.1103/PhysRevB.90.024403 (2014).

20. Takagi, R. *et al.* Spin-wave spectroscopy of the dzyaloshinskii-moriya interaction in room-temperature chiral magnets hosting skyrmions. *Phys. Rev. B* **95**, 220406, DOI: 10.1103/PhysRevB.95.220406 (2017).
